# Cross-cutting circuits in heart failure with preserved ejection fraction: Integrating adipokine biology, mechanotransduction, and precision targeting

**DOI:** 10.7150/ijbs.137328

**Published:** 2026-09-11

**Authors:** KaiJian Zhang, Hui Li, Wei Wen, BuChun Zhang

**Affiliations:** 1 Department of Cardiology, Ma'anshan People's Hospital, Wannan Medical University, Anhui Ma'anshan, 243099, China.; 2 Graduate School, Wannan Medical University, Anhui Wuhu, 241002, China.; 3 Department of Cardiology, The First Affiliated Hospital of USTC, Division of Life Sciences and Medicine, University of Science and Technology of China, Anhui Hefei, 230001, China.

**Keywords:** heart failure with preserved ejection fraction, neuroimmune regulation, macrophage heterogeneity, mechanotransduction, adipokine hypothesis

## Abstract

**Background:**

Heart failure with preserved ejection fraction (HFpEF) accounts for half of all heart failure cases. However, its complex pathophysiology, which is characterized by bidirectional interactions among immune, metabolic, and mechanical pathways, has long resisted a simple explanation.

**Approach:**

Rather than listing parallel mechanisms, this review organizes recent breakthroughs around three cross-cutting frontiers: neuroimmune regulation, cell-type-specific metabolic reprogramming, and mechanotransduction. It then integrates these frontiers within the adipokine hypothesis.

**Key findings:**

Cardiac macrophage subset imbalance, myeloid fatty acid-driven inflammation, matrix stiffness-mediated feedforward loops, and adipokine imbalance have emerged as interconnected drivers of HFpEF.

**Conclusion:**

With the aid of multi-omics and machine learning, mapping these mechanistic dialogues onto phenotype-targeted precision therapy provides a roadmap for transforming HFpEF from a syndrome defined by ejection fraction into molecularly tractable endotypes.

## 1. Introduction

Heart failure with preserved ejection fraction (HFpEF) is defined by signs and symptoms of heart failure with a left ventricular ejection fraction (LVEF) ≥ 50%, accompanied by objective evidence of diastolic dysfunction or elevated filling pressures 1. HFpEF now accounts for approximately half of all heart failure cases, and its prevalence is rising due to aging populations and the epidemics of obesity, diabetes, and hypertension 2. The annual mortality rate after hospitalization for HFpEF approaches 30%, similar to that of heart failure with reduced ejection fraction (HFrEF) 3. Despite this substantial burden, effective treatments have historically been scarce; only sodium-glucose cotransporter 2 (SGLT2) inhibitors have achieved guideline-recommended status across all ejection fraction categories 4. The evolving clinical burden has spurred technological and pathophysiological advances, as recently reviewed in comprehensive frameworks integrating diagnostic tools and disease heterogeneity 5,6.

The conceptual understanding of HFpEF has evolved significantly over the past decade. HFpEF was initially conceptualised as passive diastolic dysfunction, a mechanical consequence of left ventricular (LV) stiffness. However, contemporary understanding of the condition has evolved to recognise it as a syndrome arising from the convergence of multiple comorbidities, including hypertension, obesity, diabetes, chronic kidney disease, and atrial fibrillation 7. A subsequent paradigm shifts highlighted systemic inflammation, particularly coronary microvascular endothelial inflammation triggered by cardiometabolic comorbidities, as a central pathogenic driver. More recently, the adipokine hypothesis, first proposed by Packer and expanded in a review, has offered a unifying framework 8. According to this hypothesis, HFpEF arises primarily from the expansion and dysfunctional transformation of visceral adipose tissue, leading to altered secretion of adipokines. These changes cause systemic inflammation, plasma volume expansion, cardiac hypertrophy, and fibrosis. Adipokines are categorized into three functional domains: Domain I (cardioprotective, e.g., adiponectin, omentin-1) suppressed in excess adiposity; Domain II (upregulated as an inadequate compensatory response); and Domain III (proinflammatory, prohypertrophic, profibrotic, and antinatriuretic) 8. Thus, HFpEF results from an adiposity-driven imbalance that promotes Domain III signaling while suppressing Domain I cardioprotection. This evolving trajectory has enriched our understanding but has also produced a fragmented landscape of reviews that list mechanisms in parallel. A review of HFpEF studies published between 2024 and 2026 reveals a predominant pattern. These studies tend to include sections on inflammation (e.g., cytokines, macrophages, and T cells), metabolic dysfunction (e.g., mitochondrial biology, fatty acid oxidation, and ketone metabolism), oxidative stress, neurohormonal activation, endothelial dysfunction, and fibrosis. The studies conclude with a survey of drug classes and their associated pharmacotherapies 9,10. Although this parallel architecture is comprehensive, it obscures two critical dimensions. First, it flattens causal hierarchies, blurring the distinction between proximal drivers and downstream effectors. As a result, a circulating adipokine signal and a terminal fibrotic response are presented with equal weight. Second, it reduces cross-coupling among mechanisms. Cross-coupling is defined as the bidirectional, context-dependent interactions among immune, metabolic, and mechanical pathways that collectively drive HFpEF pathogenesis.

This review takes a different organizational approach. Rather than listing mechanisms in parallel, we focus on three interconnected areas in which significant breakthroughs have occurred over the past two years: (1) neuroimmune regulation and cardiac macrophage subset dynamics, (2) cell-type-specific metabolic reprogramming, and (3) mechanotransductive signaling coupled with matrix remodeling. For each frontier, we critically evaluate recent evidence, distinguish between established findings and controversies, and map out the causal linkages. Then, we integrate these mechanisms within the adipokine framework, which provides a unifying endocrine logic that links visceral adipose dysfunction to all three domains. Finally, we translate this integrated model into a precision treatment paradigm targeting phenotypes, drawing on advances in multi-omics profiling and machine learning(ML)-based phenotyping that are beginning to transform HFpEF into molecularly tractable endotypes.

Our review is different from other contributions in six ways: (i) It focuses on the most advanced and recent advancements in mechanistic frontiers; (ii) It organizes discussions around cross-cutting dialogues instead of simply listing them; (iii) It critically evaluates therapeutic mechanisms, differentiating between claims that are well-established and those that are speculative; (iv) It clearly links mechanisms to therapeutic targets within a framework that considers both directions; (v) It introduces mechanotransduction as a significant aspect that was lacking in previous HFpEF reviews; and (vi) It ends with a plan for moving forward to the clinic that goes beyond simply listing drugs to address how trials should be designed based on mechanisms.

## 2. Frontier I: Neuroimmune regulation and cardiac macrophage subset dynamics in HFpEF

### 2.1 Cardiac macrophage heterogeneity: from Bulk descriptions to subset-specific functions

A type of immune cell called "cardiac macrophages" has become a key player in the development of HFpEF. However, scientists have only recently figured out how these cells work, which is important for developing new treatments. There are two types of resident macrophages in the heart. The first type comes from the heart during a person's early development. These cells can reproduce on their own in the heart. The second type comes from another type of cell in the body called monocytes. These monocyte-derived macrophages leave the bone marrow and enter the heart in response to signals that cause inflammation 11,12. In the steady state, resident macrophages primarily contribute to cardioprotection, tissue homeostasis, electrical conduction, and the clearance of apoptotic cells. However, in HFpEF, this balance is disrupted by the expansion of proinflammatory monocyte-derived subsets and the contraction of reparative resident populations.

A key study by Gutiérrez et al. provided a detailed characterization of the time course of these changes in a mouse model of HFpEF (high-fat diet plus L-NAME(NG-nitro-L-arginine methyl ester)) 13. A notable shift in myocardial macrophage populations was detected as early as five weeks, during the preclinical phase before overt HFpEF manifested. Specifically, resident reparative CCR2⁻MHCII⁻ macrophages decreased while proinflammatory CCR2⁺MHCII⁺ macrophages increased. These early changes were associated with elevated circulating tumor necrosis factor alpha (TNF-α), decreased cardiac AMP-activated protein kinase (AMPK) activation, and more severe myocardial fibrosis at the onset of the disease (15 weeks).

Three implications are crucial. First, macrophage subset imbalance is not merely a consequence of established HFpEF but precedes and may promote disease development, suggesting that targeting macrophage dynamics could have preventive potential. Second, the persistence of elevated CCR2⁺MHCII⁺ macrophages at 15 weeks indicates a sustained proinflammatory signature, consistent with the chronic low-grade inflammation that characterizes human HFpEF 14. Third, the distinct trajectories of the CCR2⁻ and CCR2⁺ populations underscore the importance of therapeutic strategies that restore the balance of subsets rather than achieving complete depletion of macrophages.

One unresolved question is the relative contribution of resident versus monocyte-derived macrophages across disease stages. Gutiérrez and colleagues' temporal analysis suggests that the initial shift occurs within the resident pool, followed by monocyte recruitment and differentiation. However, lineage tracing studies with higher temporal resolution are needed to definitively determine this sequence. Additionally, the specific roles of different macrophage subsets in driving LV hypertrophy, interstitial fibrosis, and coronary microvascular dysfunction require further investigation. Recent studies using CCR2 knockout models have shown that CCR2 deletion prevents LV hypertrophy, but only partially reverses diastolic dysfunction and fibrosis. This suggests that hypertrophy and fibrosis may be driven by distinct immune mechanisms 15. This observation has direct therapeutic implications, as anti-inflammatory therapy alone may be insufficient without concurrent antifibrotic intervention.

### 2.2 Neuroimmune regulation: vagus nerve-macrophage signaling as a therapeutic node

The interface between the autonomic nervous system and cardiac immune cells has opened a new frontier of therapy. A landmark study demonstrated that transcutaneous vagus nerve stimulation (tVNS) improves the HFpEF phenotype in preclinical models by activating the α7 nicotinic acetylcholine receptor (α7nAChR) on cardiac macrophages via cholinergic signaling 16. tVNS reduced the abundance of SPP1-expressing, CCR2-positive macrophages while inducing a TLF/MHCII-positive macrophage population that expresses the pro-repair factor insulin-like growth factor 1 (IGF1). This study demonstrated that the vagus nerve-cardiac macrophage axis plays a causal role in HFpEF pathogenesis and that electrical neuromodulation can restore the balance of macrophage subsets.

From a translational perspective, tVNS has several advantages over pharmacological immunomodulation. It is noninvasive and ambulatory, and it avoids systemic immunosuppression. However, substantial challenges remain. The optimal stimulation parameters (frequency, intensity, duration, and laterality) have not been systematically determined for HFpEF, and the duration of effects after stimulation cessation is unknown. Additionally, the extent to which tVNS effects are mediated through cardiac-specific versus systemic (e.g., splenic) cholinergic pathways is unclear. This information is necessary to determine the need for targeted cardiac delivery strategies.

### 2.3 Therapeutic implications of macrophage subset targeting

The framework of the macrophage subset suggests several specific therapeutic strategies. For example, CCR2 blockade directly limits the recruitment and activation of proinflammatory, monocyte-derived macrophages. Preclinical studies using CCR2 antagonists or genetic deletion have demonstrated reduced LV hypertrophy, but incomplete reversal of established fibrosis and diastolic dysfunction 15. These results suggest that CCR2⁺ macrophages are key drivers of the hypertrophic response, while other cell types, such as fibroblasts and distinct resident macrophage populations, contribute to fibrosis independently. For the design of clinical trials, this implies that combination therapy targeting both CCR2 signaling and fibrotic pathways may be necessary, similar to the multi-target strategies that have been successful in HFrEF.

Activation of the insulin-like growth factor 1 (IGF1) pathway is a second potential strategy. tVNS-induced TLF⁺/MHCII⁺ macrophages express IGF1, and IGF1 signaling promotes tissue repair 16. However, systemic administration of IGF1 carries oncogenic and metabolic risks, and cardiac-specific delivery is not yet clinically available. α7nAChR agonists are a potential pharmacological alternative to tVNS. Several selective agonists are in clinical development for inflammatory conditions. Evaluating these agonists in HFpEF would require demonstrating cardiac macrophage modulation in humans, which is feasible through analysis of circulating monocyte phenotypes as a surrogate. A recent systematic review concluded that, although no immunotherapy has been approved for HFpEF, interventions that modulate inflammation (e.g., interleukin-1 [IL-1] blockade, mast cell stabilization, and myeloid-targeted therapies) show promise 17. The review emphasized that future clinical trials must incorporate immune profiling for patient stratification because not all HFpEF patients exhibit the same pattern of immune activation. The principle of immune endotyping will be essential as immunomodulatory approaches move toward clinical testing.

## 3. Frontier II: cell type-specific metabolic reprogramming and its therapeutic implications

### 3.1 The paradox of cardiomyocyte metabolism in HFpEF versus HFrEF

Metabolic dysregulation is a hallmark of HFpEF, but it differs fundamentally in nature from HFrEF, with significant therapeutic implications. In HFrEF, the failing heart exhibits a well-characterized shift from fatty acid oxidation (FAO) to increased glucose utilization, resembling a fetal metabolic pattern. This shift is initially compensatory, but it ultimately contributes to energetic inefficiency and contractile dysfunction 18.

In contrast, emerging evidence suggests that cardiomyocytes with HFpEF exhibit sustained or heightened FAO coupled with impaired ketone body oxidation. This leads to excessive reactive oxygen species (ROS) production and myocardial stiffness 19, 20. Despite persistently elevated FAO rates, overall metabolic flexibility is compromised, resulting in an inability of the cardiomyocyte to efficiently switch between substrates in response to variations in workload or substrate availability. This loss of metabolic flexibility may be the central metabolic pathology in HFpEF.

These metabolic profiles are being increasingly validated in humans. Plasma metabolomics has identified signatures that distinguish HFpEF from HFrEF. Markers of ketogenic reprogramming are uniquely elevated in HFrEF, whereas asymmetric dimethylarginine (ADMA) is elevated in HFpEF 21.

The mechanistic basis for sustained FAO remains incompletely understood. One hypothesis implicates the persistent activation of the peroxisome proliferator-activated receptor alpha (PPARα), which is driven by elevated levels of circulating fatty acids originating from dysfunctional adipose tissue 22. Another hypothesis suggests that impaired mitochondrial quality control prevents mitochondria from adjusting their substrate oxidation capacity to meet demand, resulting in metabolic rigidity 23. Resolving this question requires spatial metabolomics and single-cell metabolic profiling because bulk tissue analyses cannot distinguish signals specific to cardiomyocytes from signals originating in fibroblasts, endothelial cells, or immune cells.

### 3.2 Myeloid cell fatty acid metabolism in HFpEF: connecting metabolic dysregulation to immune activation

Aberrant immune activation in HFpEF is driven by reprogrammed fatty acid metabolism in myeloid cells. A seminal study by Filipp et al. demonstrated that defective FAO in myeloid cells leads to intracellular lipid accumulation. This accumulation paradoxically activates neighboring hematopoietic stem cells, promoting the generation of pro-inflammatory monocytes and exacerbating HFpEF pathology 24. Single-cell RNA sequencing revealed that metabolic stress, particularly dyslipidemia, activates distinct populations of cardiac macrophages characterized by altered metabolic gene signatures 25. The mechanistic target of rapamycin (mTOR) pathway appears to be a key integrator of metabolic signals. Its dysregulation in macrophages exacerbates inflammatory responses in cardiovascular diseases, including HFpEF 26. Furthermore, S100A9, a molecule induced by metabolic stress that modulates mitochondrial fission and oxidative stress, has been shown to be pathogenic. Inhibiting S100A9 ameliorates HFpEF symptoms 27. Taken together, these findings, largely derived from mouse models, are supported by human data showing that circulating monocyte metabolic profiles correlate with diastolic dysfunction and HFpEF status, suggesting that altered fatty acid handling in myeloid cells actively instigates the proinflammatory state rather than passively resulting from it.

Metabolically reprogrammed myeloid cells perpetuate the primary characteristics of HFpEF, such as diastolic dysfunction, fibrosis, and sterile inflammation. These cells also reveal novel therapeutic vulnerabilities. Myeloid cell activation results in the release of potent pro-fibrotic and inflammatory mediators. For instance, IL-1β released by myeloid cells contributes to pulmonary hypertension in HFpEF models, and CXCR4-dependent signaling from macrophages to fibroblasts directly promotes cardiac diastolic dysfunction 28,29. This maladaptive crosstalk is amplified by the recruitment and altered function of monocyte subsets, including non-classical monocytes associated with diastolic function markers 30. Targeting myeloid metabolic pathways shows promise. The TREM2 receptor, which is involved in lipid sensing and efferocytosis, has emerged as a critical node. TREM2 agonism, such as with eicosapentaenoic acid, promotes TREM2-dependent efferocytosis and attenuates HFpEF. Furthermore, TREM2 itself is a mediator in hypertensive heart failure 31, 32. Additionally, preclinical studies have demonstrated the efficacy of strategies that modulate immune metabolic pathways, such as vagus nerve stimulation and vitamin B6 supplementation, in reversing HFpEF phenotypes 33, 34. Targeting myeloid fatty acid metabolism to restore resolution pathways, which are often defective in obesity and HFpEF, may be a fundamental strategy to break the cycle of sterile inflammation and metabolic stress 35.

### 3.3 SGLT2 inhibitors in HFpEF: mechanisms revisited

SGLT2 inhibitors are the only pharmacological class with guideline-recommended status for HFpEF, based on large randomized trials demonstrating reduced heart failure hospitalizations 36. However, the mechanisms underlying these benefits are not fully understood and are still being debated. A review critically examined whether the effects of SGLT2 inhibitors on cardiac metabolism in HFpEF are fact or fiction 37. The review concluded that, while SGLT2 inhibitors do modulate myocardial glucose utilization, FAO, and mitochondrial function in preclinical models, the evidence that these metabolic effects benefit humans is correlative rather than causal.

Several alternative mechanisms have been proposed. Hemodynamic effects include modest natriuresis and plasma volume reduction; however, the magnitude is smaller than that of loop diuretics. Renal mechanisms include preserving renal function, reducing uremic toxins, and improving erythropoietin production. Systemic metabolic effects include weight loss, improved insulin sensitivity, and reduced visceral adipose tissue. Direct cardiac effects include the inhibition of the sodium-hydrogen exchanger (NHE1) in cardiomyocytes, which reduces oxidative stress and improves mitochondrial calcium handling 38, 39.

The primary challenge in distinguishing among these mechanisms is that they are not mutually exclusive and likely operate simultaneously. However, from a drug development perspective, the distinction is significant. For example, if the primary benefit is hemodynamic, loop diuretics might achieve similar effects. If the benefit is renal, finerenone may provide additive or overlapping benefits 40. If the benefit is through adipose tissue remodeling, GLP-1 receptor agonists (GLP-1 RAs) may target the same pathway. Understanding the predominant mechanism in specific patient subgroups could facilitate more rational combination therapy and patient selection. For instance, it could clarify whether SGLT2 inhibitors primarily reduce adipose tissue in obese patients or protect the kidneys in patients with chronic kidney disease 41.

### 3.4 GLP-1 receptor agonists: beyond weight loss

GLP-1 RAs have emerged as a transformative therapeutic class for obesity-related HFpEF. The STEP HFpEF trial program demonstrated that semaglutide significantly improved symptoms, functional capacity, and quality of life in obese patients with HFpEF. The treatment produced a 13.3% reduction in body weight and increased the 6-minute walk distance by 21.5 meters 42, 43.

Tirzepatide, a dual GLP-1/glucose-dependent insulinotropic polypeptide (GIP) receptor agonist, produced a greater weight reduction of 13.9% and improved six-minute walk distance by 26 meters. It also resulted in a marked reduction in systemic inflammation, with a 43.5% decrease in C-reactive protein 44. Notably, tirzepatide significantly reduced the risk of cardiovascular death and worsening heart failure events in patients with obesity-related HFpEF 45.

A critical question is whether the benefits of GLP-1 RAs in HFpEF are solely mediated through weight loss or if they have direct cardiac effects that contribute independently. Secondary analyses of the STEP HFpEF trial suggest that the beneficial effects extend beyond weight loss and include direct anti-inflammatory and anti-fibrotic cardiac effects 46. Preclinical studies support this interpretation, demonstrating that low-dose GLP-1 RA therapy exerts anti-fibrotic effects across multiple organs in cardiometabolic HFpEF, independent of weight change 47,48.

However, limitations must be acknowledged. Current trial durations are relatively short (≤52 weeks), and the long-term effects on hard cardiovascular endpoints, including cardiovascular and all-cause mortality, are uncertain. A pooled analysis confirmed improvements in symptoms and functional capacity, yet it failed to demonstrate a reduction in heart failure hospitalizations 49. This contrasts with SGLT2 inhibitors, which consistently reduce the risk of hospitalization, suggesting that GLP-1 RAs primarily improve quality of life and functional capacity. Whether longer follow-up will reveal hospitalization benefits remains to be determined. Furthermore, trial populations are limited in diversity, and data on non-obese HFpEF patients are scarce.

The cell type-specific metabolic reprogramming in HFpEF and therapeutic nodes is illustrated in **Figure 1**.

## 4. Frontier III: mechanotransductive signaling, matrix remodeling, and cardiac stiffness

### 4.1 Mechanical forces as pathogenic drivers: the mechanobiology of HFpEF

Cardiac stiffness, the hallmark mechanical abnormality in HFpEF, is fundamentally a mechanobiology problem. Increased LV stiffness results from passive factors, such as extracellular matrix (ECM) composition and cross-linking, and titin isoform shifts, as well as active factors, such as impaired relaxation due to sarcoplasmic reticulum calcium handling defects 50. However, the relationship between mechanical forces and cellular behavior is bidirectional. Altered mechanics result from cellular dysfunction and also feedback to modulate cellular phenotype through mechanotransduction signaling pathways 51.

In a healthy heart, cardiomyocytes and fibroblasts sense mechanical forces through multiple mechanosensors, including integrins (which link the ECM to the cytoskeleton), stretch-activated ion channels (e.g., transient receptor potential [TRP] channels), and transcriptional regulators, such as Yes-associated protein (YAP) and transcriptional coactivator with PDZ-binding motif (TAZ) 52, 53. In HFpEF, increased diastolic stiffness creates elevated baseline mechanical stress, which activates mechanosensors that drive pathological gene expression programs. YAP/TAZ signaling is particularly relevant; under increased mechanical stress, YAP/TAZ translocate to the nucleus and co-activate transcription factors, including TEAD (TEA domain family member), thereby promoting cardiomyocyte hypertrophy, fibroblast proliferation, and ECM deposition 54. Notably, Yap/Taz activation interacts with metabolic regulation. Yap promotes glycolysis and suppresses FAO, which is the opposite pattern observed in HFpEF cardiomyocytes 55. This apparent contradiction suggests that distinct metabolic outcomes are determined by the timing of Yap/Taz activation (early versus late), or that Yap/Taz signaling may be differentially regulated in HFpEF versus other cardiac pathologies. Studies of human myocardial tissue have confirmed increased nuclear YAP/TAZ in HFpEF samples, supporting the relevance of these mechanosensitive pathways 56.

### 4.2 Matrix remodeling: from ECM composition to stiffness-mediated feedforward loops

Myocardial fibrosis is a nearly universal feature of HFpEF, present in approximately 50% of patients as assessed by cardiovascular magnetic resonance (CMR) with extracellular volume mapping 9. Increased collagen deposition leads to increased myocardial stiffness, which elevates LV filling pressures and impairs exercise tolerance. However, the relationship between fibrosis and diastolic dysfunction is complex. The quality of the ECM, particularly the cross-linking of collagen facilitated by lysyl oxidase (LOX), may be as important as the amount of collagen 57.

One critical yet underappreciated dimension is the feed-forward loop, whereby increased matrix stiffness itself promotes further fibrosis. Compared to cells on normal stiffness matrices (~5-15 kPa), cultured cardiac fibroblasts seeded on hydrogels with stiffness matching fibrotic myocardium (~50-100 kPa) exhibit increased proliferation, transforming growth factor beta (TGF-β) activation, and collagen synthesis 58. This stiffness-induced fibroblast activation is mediated by integrin signaling, Rho-associated protein kinase (ROCK) activation, and YAP/TAZ nuclear translocation 59. Once a threshold of matrix stiffness is exceeded, a self-perpetuating cycle ensues. Increased matrix stiffness promotes fibroblast activation and collagen deposition, which leads to further increased stiffness. This positive feedback loop has important therapeutic implications. First, intervening early, before stiffness reaches a critical threshold, may be more effective than intervening later, after the feedforward loop has been established. Second, combining antifibrotic therapy with mechanical unloading may produce synergistic effects. Third, strategies that reduce existing matrix stiffness (e.g., inhibiting LOX or activating matrix metalloproteinases [MMPs]) could potentially disrupt the feedback loop, even when collagen deposition persists.

Several anti-fibrotic agents have been evaluated for use in patients with HFpEF, but the results have been mixed. Pirfenidone, a TGF-β inhibitor approved for idiopathic pulmonary fibrosis, showed modest improvements in diastolic function in a small Phase 2 trial, but it has not been tested in larger studies 60. Galectin-3 inhibitors, which target a lectin that promotes fibroblast activation and collagen cross-linking, have shown promise in preclinical studies but have not yet demonstrated clinical benefit 61. LOX inhibitors, which prevent collagen cross-linking, offer a different approach 62. The failure of single-agent antifibrotic trials thus far may suggest that fibrosis is a downstream consequence rather than a primary driver or that combination therapy targeting multiple processes (e.g., synthesis, cross-linking, and degradation) is necessary.

### 4.3 Lessons from neutral and negative trials in fibrosis and inflammation

To provide a balanced perspective, it is important to acknowledge that several therapeutic strategies targeting fibrosis and inflammation have failed to meet primary endpoints in HFpEF. For example, the PIROUETTE trial of pirfenidone showed a reduction in myocardial fibrosis by CMR but did not translate into clear clinical benefit 60. Similarly, galectin-3 inhibition did not improve outcomes in heart failure populations despite a strong biological rationale 63. Although anti-inflammatory approaches, such as IL-1 blockade, have shown efficacy in atherosclerotic disease, they have not been tested in dedicated HFpEF outcome trials 64. Secondary analyses from canakinumab trials have not demonstrated consistent heart failure benefits 65. Potential explanations include the following: (1) patient heterogeneity, whereby fibrosis and inflammation are not uniformly active in all HFpEF patients, (2) compensatory feedback, whereby blocking a single pathway may upregulate parallel profibrotic or proinflammatory cascades, and (3) irreversibility, whereby once established, matrix cross-linking may be refractory to pharmacological reversal. These findings underscore the importance of biomarker-enriched trial designs and combination therapies that target synthetic, cross-linking, and degradative ECM processes, as well as upstream adipokine and metabolic drivers simultaneously.

### 4.4 Mechano-metabolic-immune coupling: the missing integration

The immune system, metabolism, and mechanics are the three mechanistic frontiers that are closely interconnected. There are several examples that illustrate this cross-coupling. First, matrix stiffness directly influences the phenotype of macrophages. Macrophages grown on stiff matrices become pro-inflammatory (M1-like) and produce TNF-α and IL-6, whereas soft matrices promote reparative (M2-like) polarization 66. in HFpEF, increased myocardial stiffness may directly polarize infiltrating macrophages toward a proinflammatory state, creating a convergence of mechanical and immune pathology. Second, metabolic intermediates regulate mechanotransduction. Succinate accumulates under conditions of mitochondrial dysfunction and stabilizes hypoxia-inducible factor 1α (HIF-1α), thereby promoting YAP activation 67. This provides a mechanism whereby metabolic dysregulation directly activates the YAP/TAZ mechanotransductive pathway, linking metabolic pathology to mechanical outcomes. Third, immune-derived cytokines modulate matrix stiffness. Proinflammatory cytokines, such as IL-1β and TNF-α, initially degrade the ECM by upregulating MMP expression. However, persistent signaling leads to dysregulated repair and aberrant collagen deposition 68. Conversely, TGF-β, which is derived from macrophages, promotes the differentiation of fibroblasts into myofibroblasts and collagen synthesis 69.

These cross-couplings strongly support an integrated view of HFpEF pathogenesis rather than a reductionist model based on a single primary mechanism. Next, we will discuss the adipokine hypothesis, which explains how adipose tissue dysfunction can influence all three mechanisms simultaneously.

Figure 2 displays the cross-coupling mechanisms among the neuroimmune, metabolic, and mechanotransductive pathways in HFpEF.

## 5. The adipokine hypothesis as an integrative framework

Proposed by Packer in 2025, the adipokine hypothesis posits that HFpEF primarily arises from the expansion and dysfunction of visceral adipose tissue. This tissue secretes an altered suite of adipokines that drive systemic inflammation, volume expansion, cardiac hypertrophy, and fibrosis 8. The hypothesis is supported by nine lines of evidence, which include the following: (1) obesity and dietary nutrient excess are major drivers of experimental HFpEF; (2) changes in visceral adiposity and circulating adipokines predict HFpEF years in advance; (3) central obesity is present in over 95% of HFpEF patients; (4) obesity and HFpEF exhibit striking parallelism in molecular and clinical features; (5) characteristic adipokine changes correlate with disease severity; (6) adipokines have well-established effects on cardiac structure and function; (7) bariatric surgery and pharmacotherapies that reduce visceral fat improve HFpEF outcomes; (8) excess adiposity identifies patients most likely to respond to current treatments; and (9) experimental interventions that target adipokine secretion modulate cardiac pathology 70-72.

The adipokine framework integrates three mechanistic frontiers. Domain III adipokines, such as leptin, resistin, and visfatin, promote proinflammatory macrophage polarization. This directly links adipose dysfunction to immune pathology 73, 74. Fatty acid binding protein 4 (FABP4), another Domain III adipokine, modulates fatty acid metabolism in adipocytes and myeloid cells, connecting adipose dysfunction to metabolic reprogramming 75. Leptin directly affects cardiac fibroblasts, promoting collagen synthesis and matrix stiffening through YAP/TAZ activation and bridging adipose signaling to mechanotransduction 76.

It is important to note that the adipokine hypothesis does not claim an absolute correlation between obesity and HFpEF. Approximately 20-30% of HFpEF patients have a normal BMI. However, even non-obese HFpEF patients often exhibit increased visceral adiposity that is disproportionate to their BMI, or altered adipokine profiles, without overt obesity 8, 77. For example, increased thickness of epicardial adipose tissue and an elevated leptin-to-adiponectin ratio have been documented in non-obese HFpEF patients, indicating a state of adipose tissue dysfunction that is independent of total body weight 78. This nuance is critical for clinical translation. The relevant target is the biology and distribution of adipose tissue, not merely body weight. Therefore, therapeutic strategies should focus on normalizing adipokine profiles instead of just reducing weight.

The adipokine framework helps to explain the efficacy of SGLT2 inhibitors and GLP-1 RAs beyond their effects on lowering glucose and promoting weight loss. Both drug classes reduce visceral adipose tissue mass and improve adipose tissue function. This shifts the adipokine balance toward the suppression of Domain III adipokines and the restoration of Domain I cardioprotective factors 79, 80. We hypothesize that this common downstream pathway of adipose tissue remodeling is why mechanistically distinct drugs converge on similar clinical benefits.

## 6. Integration: toward phenotype-targeted precision therapy

### 6.1 From mechanism modules to therapeutic nodes

The preceding sections identified multiple therapeutic nodes within each mechanistic frontier. Table 1 provides a systematic mapping of mechanisms to targets to therapeutic strategies, including the current level of evidence for each approach.

### 6.2 Biomarker-driven phenotyping for treatment allocation

The heterogeneity of HFpEF has persistently challenged therapeutic development. Landmark trials in unselected HFpEF populations often failed to demonstrate a therapeutic benefit. However, subsequent analyses revealed that the benefit was concentrated in specific subgroups, most notably patients with obesity or metabolic syndrome. These findings have prompted intensive efforts to identify molecular and clinical phenotypes that predict differential treatment responses.

Multi-omics approaches have emerged as powerful tools for phenotyping. A systematic review and meta-analysis of multi-omics biomarkers in heart failure (HF; 28 studies, various HF phenotypes) reported a pooled standardized mean difference of 0.273 (95% confidence interval [CI]: 0.226, 0.320) and a combined hazard ratio of 1.28 (95% CI: 1.10, 1.48) for adverse outcomes 81. Transcriptomic analyses revealed key gene expression patterns implicating inflammation and ECM remodeling. Proteomic data highlighted biomarkers of mitochondrial dysfunction, and metabolomic profiles identified metabolic shifts correlated with therapy responsiveness 82.

Specifically for HFpEF, plasma metabolomics has identified distinct signatures that differentiate HFpEF from HFrEF. Naeem et al. analyzed 90 metabolites in 787 samples and found that asymmetric dimethylarginine (ADMA) was uniquely elevated in HFpEF. In contrast, markers of ketogenic metabolic reprogramming, such as 3-hydroxybutyrate and its metabolite C4-OH carnitine, were uniquely elevated in HFrEF 22. These findings suggest that circulating metabolites could serve as phenotype-specific biomarkers to guide therapy allocation.

Machine learning (ML) has further advanced precision phenotyping. It has enabled genome-wide association studies that have revealed the genetic architecture of HFpEF with unprecedented clarity. Similarly, ML-based multi-omics integration has been shown to detect and characterize HFpEF phenotypes years before the onset of clinical symptoms, when the disease's trajectory may still be modifiable 83. A recent study showed that ML-based phenotyping can identify HFpEF clusters with different clinical characteristics, prognostic trajectories, and drug responses 84.

### 6.3 Clinical trial design for the precision era

The advent of multiple effective therapies for HFpEF, such as SGLT2 inhibitors, finerenone, and GLP-1 receptor agonists, has transformed the therapeutic landscape, but it has also created new challenges for trial design. Key questions remain.

#### 6.3.1 Combination therapy sequencing

Although at least three drug classes have demonstrated benefits, the optimal sequencing and combination strategies remain unknown. The FINEARTS HF trial confirmed finerenone's effectiveness in HFpEF patients, significantly reducing their risk of cardiovascular death or hospitalization for heart failure 85. Finerenone reduced morbidity and mortality consistently across a broad range of glycemic statuses and glucose-lowering regimens, including in patients who were already receiving SGLT2 inhibitors or GLP-1 RAs 86. However, adequately powered trials have not yet formally tested the additional benefits of combining finerenone with SGLT2 inhibitors or GLP-1 RAs.

#### 6.3.2 Enrichment strategies

Since HFpEF therapies provide different benefits to different patient subgroups, future trials should use enrichment designs that select patients based on the most relevant mechanism of the investigational agent. For anti-inflammatory strategies, for example, enrichment based on elevated C-reactive protein (CRP) or specific cytokine profiles would increase statistical power. For anti-fibrotic agents, enrichment based on evidence of diffuse fibrosis from CMR (e.g., elevated extracellular volume) would be appropriate. For GLP-1 RAs, enrichment based on obesity or metabolic syndrome is already standard.

#### 6.3.3 Endpoint selection

The discrepancy between functional improvement with GLP-1 receptor agonists and reduced hospitalization with SGLT-2 inhibitors and finerenone raises questions about the optimal endpoints for HFpEF trials. To capture the full spectrum of treatment benefit, a dual primary endpoint approach with co-primary endpoints of cardiovascular death or heart failure hospitalization and a patient-reported outcome, such as the Kansas City Cardiomyopathy Questionnaire (KCCQ) score, may be necessary.

#### 6.3.4 Real-world evidence integration

The favorable safety profiles of SGLT2 inhibitors, finerenone, and GLP-1 RAs in clinical trials have led to their rapid adoption in clinical practice. Registry-based randomized trials and pragmatic designs can generate real-world evidence on comparative effectiveness and long-term safety, thereby complementing traditional randomized controlled trials (RCTs).

Figure 3 provides a schematic illustration of the mechanism-to-precision therapy mapping in HFpEF.

### 6.4 Emerging therapeutic strategies on the horizon

Several novel strategies are in the preclinical or early clinical development stages. These include gene therapy approaches targeting sarcomeric proteins, such as titin splicing modulation to reduce passive stiffness, and calcium handling proteins, such as SERCA2a overexpression. Cell therapy using cardiac progenitor cells or mesenchymal stromal cells has shown mixed results, though it may benefit certain subgroups 87. Non-coding RNA therapeutics that target microRNAs involved in fibrosis (e.g., miR-21 and miR-29) or hypertrophy (e.g., miR-208) are in preclinical development 88. Immunomodulatory biologics that target specific cytokines (IL-1β, IL-6, and TNF-α) have shown promise in heart failure, though they have yet to be systematically evaluated in HFpEF patients 89, 90. Each strategy should be matched to the patient phenotype most likely to respond, guided by the mechanistic framework developed above.

### 6.5 Implementation challenges for precision cardiology in HFpEF

Several substantial barriers stand in the way of translating the promise of multi-omics and machine learning-based precision therapy into clinical practice. First are cost and infrastructure. High-throughput proteomics, metabolomics, and genomic sequencing, combined with advanced imaging and computational pipelines, require investments that are currently concentrated in specialized academic centers. Widespread adoption will require scalable, cost-effective assays and cloud-based analytical platforms. Second is access and equity. The populations bearing the highest HFpEF burden, including older adults, racial and ethnic minorities, and those in low-resource settings, are often underrepresented in omics studies. These populations may also face disparities in access to precision diagnostics. Third, there is the issue of regulatory and evidentiary proof. Biomarker-guided trials must validate their analytical and clinical accuracy and confirm clinical utility, meaning better patient outcomes than standard care, before they are accepted by payers and guideline panels. Overcoming these challenges will require collaborative networks, pragmatic trial designs embedded in electronic health records, and policy frameworks that incentivize developing accessible precision tools.

## 7. Outstanding questions and future directions

**Despite substantial progress, several fundamental questions remain. We identify five priority areas for future investigation.**Question 1: What is the causal hierarchy among mechanisms? **Does adipose dysfunction precede immune activation, or do systemic inflammatory signals cause adipose dysfunction? Does mechanical stiffness arise secondary to fibrosis, or does mechanotransduction signaling initiate fibrosis independently? Resolving these causal relationships requires multi-omics time-series data from preclinical models and human cohorts with longitudinal sampling before and after disease onset. Causal inference methodologies, including Mendelian randomization with genetic instruments for adiposity, inflammatory markers, and ECM proteins, could help determine directionality.**

Question 2: Can cell type-specific targeting be achieved clinically? **The observation that myeloid-specific deletion of CD36 recapitulates the benefits of a global metabolic intervention suggests that targeting specific cell types could achieve the same efficacy with reduced off-target toxicity. However, clinically available delivery systems for cell-type-specific targeting in the heart are limited. Lipid nanoparticles and adeno-associated virus vectors with cell-type-specific tropism are under active development and could enable macrophage- or fibroblast-directed therapies within the next decade.**

Question 3: Do existing animal models sufficiently recapitulate human HFpEF? **The most frequently used heart failure with preserved ejection fraction (HFpEF) models include a high-fat diet plus L-NAME, ZSF1 obese rats, and aging female Dahl salt-sensitive rats. While these models successfully replicate certain HFpEF characteristics, they cannot fully reproduce the full array of human comorbidities and treatment responses. Human induced pluripotent stem cell (iPSC)-derived cardiomyocyte-fibroblast-macrophage co-culture systems and organ-on-chip platforms incorporating mechanical loading may provide complementary platforms for mechanistic analysis and drug screening.**

Question 4: Which biomarkers are ready for clinical implementation? **Although numerous candidate biomarkers have been identified through omics approaches, none have been prospectively validated in large, multicenter cohorts for treatment allocation. A priority is to conduct biomarker-stratified trials, in which patients are randomized based on their biomarker status (e.g., elevated ADMA, a specific adipokine profile, or a fibrosis imaging biomarker), to determine if outcomes improve with biomarker-guided therapy compared to unguided therapy.**

Question 5: When will precision therapy for HFpEF become clinical reality? **The convergence of multiple validated therapies, a unifying mechanistic framework (the adipokine hypothesis), and powerful tools for identifying subtypes (multi-omics and ML) suggests that the first trials focusing on specific, molecularly defined subgroups will be conducted within the next five years. However, this progress will require substantial investment in biobanking, high-throughput molecular profiling, and adaptive platform trial infrastructure. The HFpEF field is poised to become a proving ground for precision cardiology, where therapies are matched to molecularly defined disease programs rather than left ventricular ejection fraction (LVEF) cutoffs alone.**

## 8. Conclusions

HFpEF has transitioned from a poorly understood, treatment-resistant syndrome to a condition with multiple emerging therapeutic options and a mechanistic basis. This transformation has been driven by advances in three interconnected areas: neuroimmune regulation, cell-type-specific metabolic reprogramming, and mechanotransduction signaling. Each of these areas has yielded testable therapeutic hypotheses and, in some cases, clinically validated interventions. The adipokine hypothesis provides a unifying framework that integrates these mechanisms. This framework positions visceral adipose tissue dysfunction as the proximal driver and adipokine signaling as the critical mediator that links obesity to cardiac pathology.

The therapeutic landscape for HFpEF has fundamentally changed with the demonstration that SGLT2 inhibitors, finerenone, and GLP-1 Ras improve clinically meaningful outcomes. The challenge now is to transition from a one-size-fits-all approach to phenotype-targeted, precision therapy guided by multi-omics biomarkers and ML-based phenotyping. These tools can identify which patients are most likely to benefit from a given intervention, either alone or in combination, while also recognizing the hurdles to implementation that must be overcome.

This review provides a roadmap for the next phase of HFpEF research by prioritizing cross-cutting mechanistic dialogues over parallel enumeration. By focusing on the interactions among immune, metabolic, and mechanical pathways and grounding these interactions in the adipokine framework, we aim to accelerate the translation of mechanistic insights into clinical practice. Over the next decade, we will discover if precision cardiology for HFpEF will fulfill its promise of transforming a syndrome defined by ejection fraction into a set of molecular endotypes with targeted therapies.

## Figures and Tables

**Figure 1:**
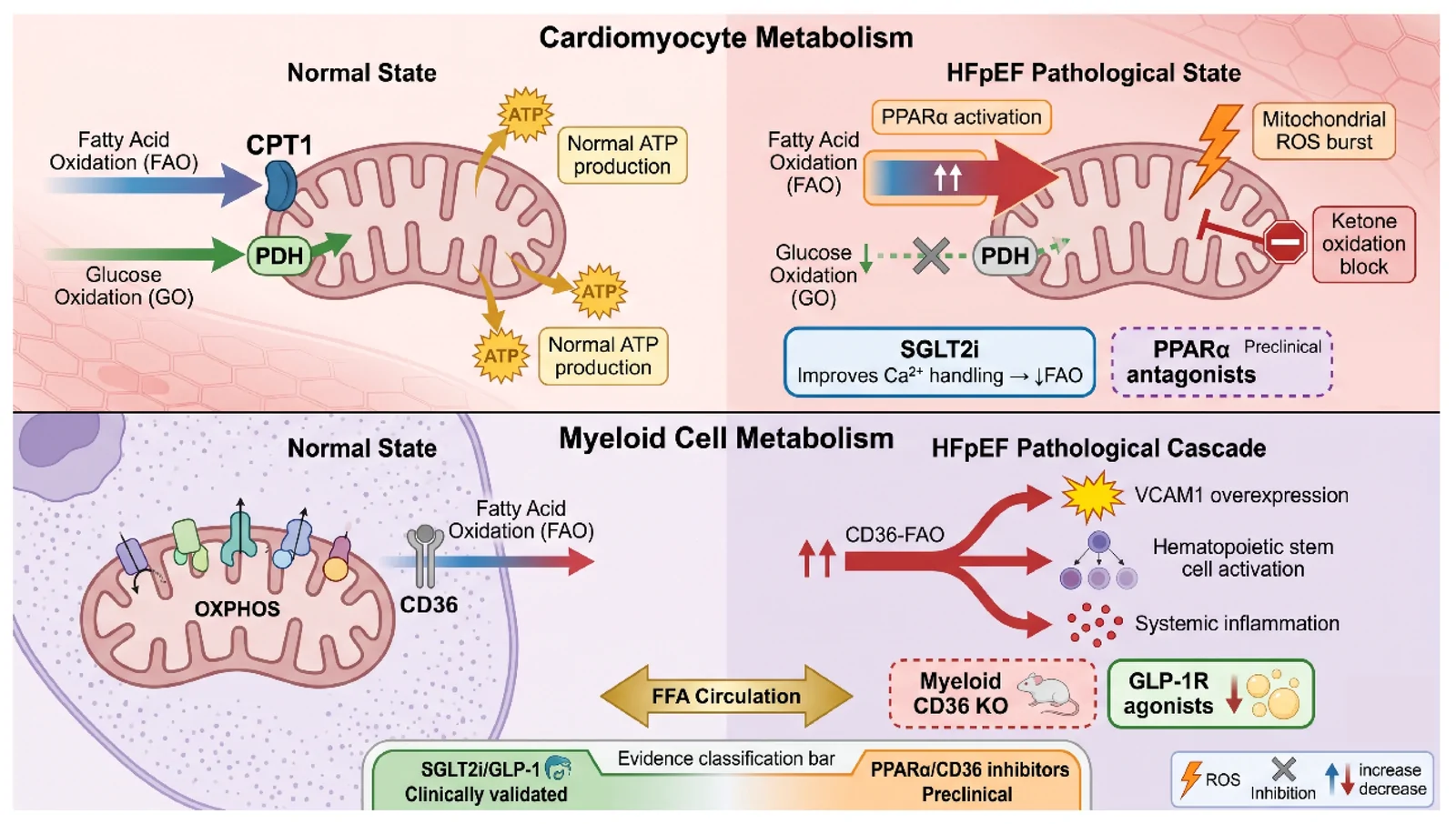
** Cell type-specific metabolic reprogramming in HFpEF and therapeutic nodes.** Cardiomyocytes (upper panel) exhibit sustained fatty acid oxidation (FAO) and impaired ketone oxidation. This increases reactive oxygen species (ROS) and myocardial stiffness. Myeloid cells (lower panel) upregulate CD36-mediated FAO, which leads to vascular cell adhesion molecule 1 (VCAM-1) expression, hematopoietic stem cell activation, and systemic inflammation. Circulating free fatty acids (FFA) from dysfunctional adipose tissue drive both processes. Therapeutic interventions are positioned at corresponding nodes: SGLT2 inhibitors and PPARα antagonists target cardiomyocyte metabolism, while myeloid-specific CD36 inhibitors and GLP-1 receptor agonists target myeloid cell metabolism by reducing lipolysis. Evidence levels are indicated by border colors. The border colors denote the translational stage of the corresponding therapies based on current human evidence: green indicates established clinical evidence (Phase 3 trials or large-scale clinical studies); orange indicates preclinical evidence; and gray represents exploratory or limited early-phase clinical evidence.

**Figure 2:**
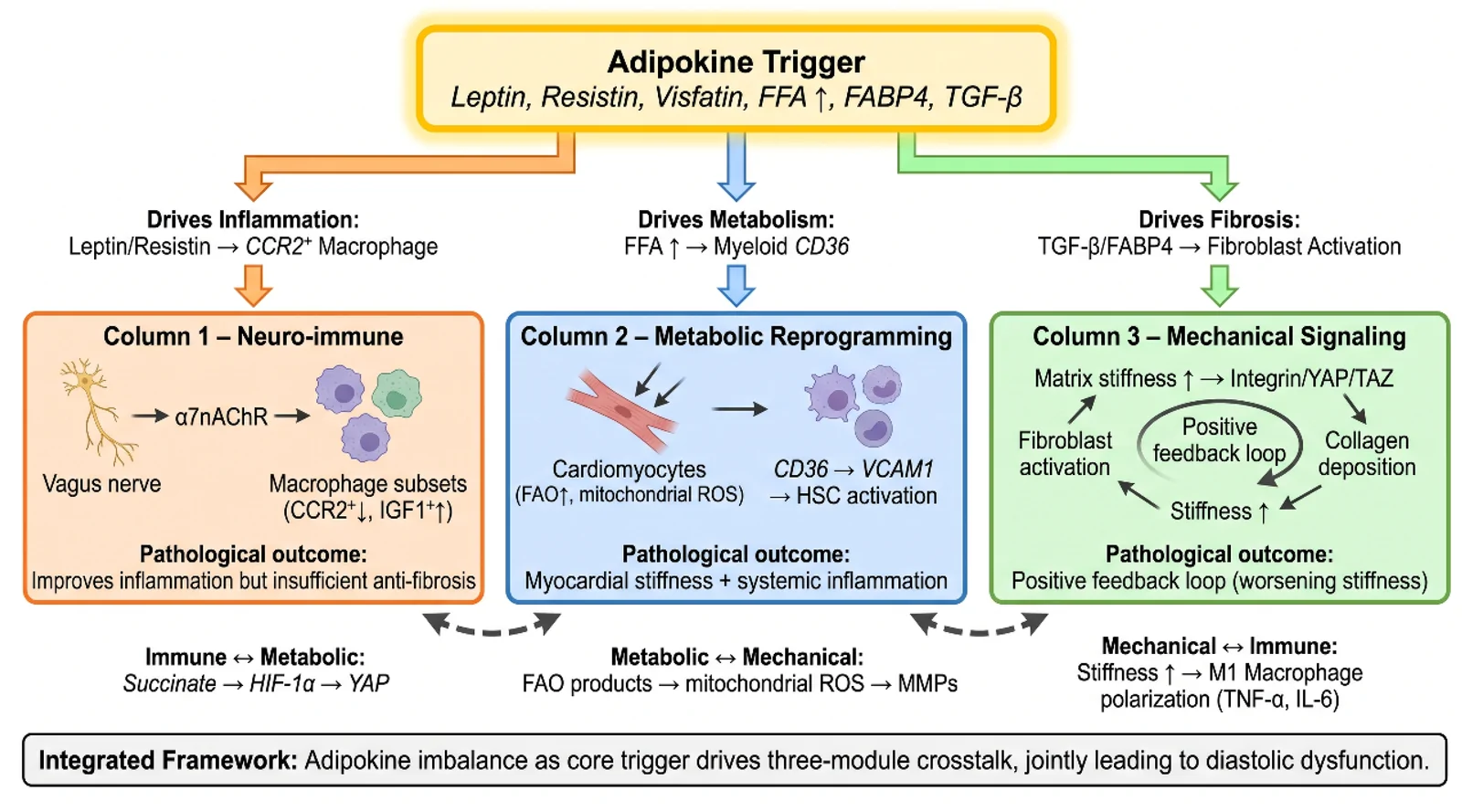
** Mechanistic cross-coupling among neuroimmune, metabolic, and mechanotransductive pathways in HFpEF**. Visceral adipose dysfunction drives three core mechanistic modules via adipokines and free fatty acids. Each module exhibits internal positive feedback loops, depicted within each column. Bidirectional arrows between modules highlight key molecular intersections: the immune-metabolic intersection (succinate-HIF-1α-YAP), the metabolic-mechano intersection (ROS-mediated MMP modulation), and the mechano-immune intersection (stiffness-induced M1 macrophage polarization). The integrated framework (bottom) positions adipokine imbalance as the trigger that sets off a self-perpetuating network that ultimately results in diastolic dysfunction. In this framework, dysfunctional visceral adipose tissue serves as the primary upstream trigger, releasing adipokines that initiate and propagate the neuroimmune, metabolic, and mechanotransductive maladaptive circuits.

**Figure 3:**
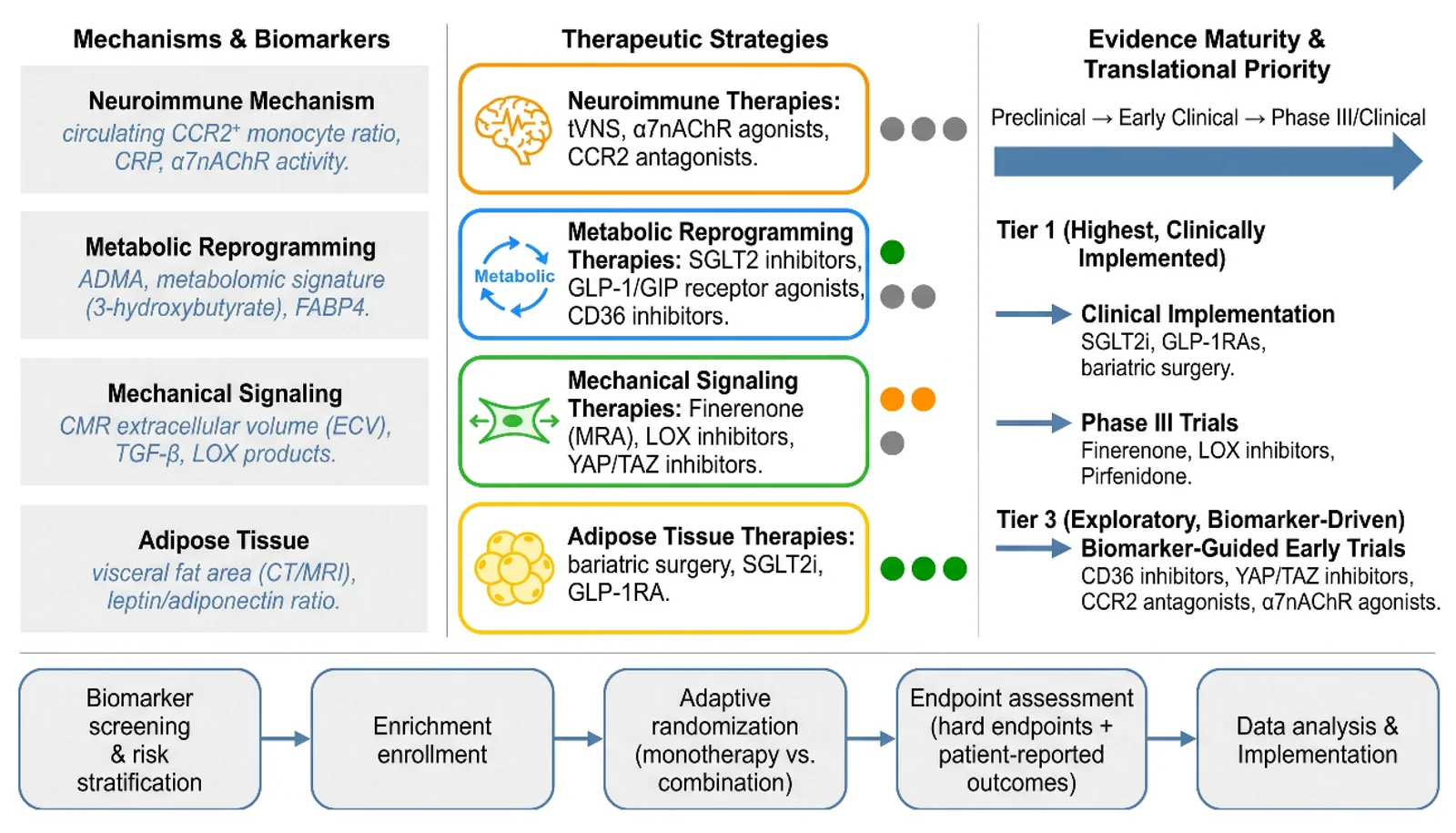
** Mechanistic mapping and translational prioritization in HFpEF.** The left panel illustrates the primary pathophysiological modules and their corresponding circulating or imaging biomarkers. The middle panel maps these mechanisms to candidate therapeutic strategies. The right upper panel indicates current evidence levels (green, established Phase 3; orange, Phase 2; gray, preclinical). The right lower panel presents a translational priority timeline, categorizing the strategies into three queues based on their readiness for clinical testing: Queue 1 (clinically ready), Queue 2 (large-scale trials underway), and Queue 3 (requiring biomarker-driven early-phase validation). The bottom panel outlines a proposed adaptive clinical trial design integrating biomarker enrichment and combined therapy endpoints.

**Table 1:** Mechanism-to-target mapping in HFpEF

Mechanism module	Key molecular pathways	Candidate therapeutic strategies (Ref)	Evidence level	Human evidence available
Neuroimmune	CCR2⁺ macrophage recruitment	CCR2 antagonists 13,17-18	Preclinical	No
	α7nAChR signalling	α7nAChR agonists, tVNS 16	Preclinical	No
	IGF1 pathway	IGF1 analogs (cardiac-targeted) 18	Exploratory	Limited
Metabolic	Myeloid CD36-FAO	CD36 inhibitors, SGLT2 inhibitors (indirect) 20	Preclinical/clinical	Yes
	Cardiomyocyte FAO	PPARα antagonists, mitochondrial modulators 24,25	Exploratory	Limited
	GLP-1/GIP signalling	Semaglutide, tirzepatide 44,45	Phase 3	Yes
Mechanotransductive	YAP/TAZ-TEAD	YAP/TAZ inhibitors 55-58	Preclinical	No
	TGF-β/Smad	Pirfenidone, galectin-3 inhibitors 63,64	Phase 2	Limited
	LOX-mediated cross-linking	LOX inhibitors 69	Preclinical	No
Adipokine	Domain III adipokines	SGLT2 inhibitors, GLP-1 RAs 84,85	Clinical	Yes
	Visceral adipose tissue	Bariatric surgery 77	Clinical	Yes

HFpEF, heart failure with preserved ejection fraction; CCR2, C-C chemokine receptor type 2; α7nAChR, α7 nicotinic acetylcholine receptor; tVNS, transcutaneous vagus nerve; IGF1, insulin-like growth factor I; FAO, fatty acid oxidation; SGLT2, sodium-dependent glucose transporters 2; PPARα, peroxisome proliferator-activated receptor alpha; GLP-1, glucagon-like peptide-1; GIP, glucose-dependent insulinotropic polypeptide; YAP, yes-associated protein; TAZ, transcriptional coactivator with PDZ-binding motif; TEAD, TEA domain transcription factor; TGF-β, transforming growth factor beta; LOX, lysyl oxidase.
